# Orbital Exenteration in Maxillary Osteosarcoma: A Case Report

**DOI:** 10.7759/cureus.98271

**Published:** 2025-12-01

**Authors:** Sannareddy Pavan Krishna Reddy, Stephen Sudhakar

**Affiliations:** 1 Ophthalmology, Sri Ramachandra Institute of Higher Education and Research, Chennai, IND

**Keywords:** craniofacial tumor, maxillary osteosarcoma, orbital exenteration, osteogenic sarcoma, proptosis

## Abstract

Maxillary osteosarcoma is a rare malignant bone tumor of the craniofacial skeleton, accounting for a small proportion of head and neck sarcomas. Orbital involvement is exceptionally uncommon and poses significant diagnostic and therapeutic challenges. Early recognition and multidisciplinary management are critical for optimizing outcomes. A 41-year-old male presented with progressive nasal obstruction for one month and left eye vision loss for 20 days. Examination revealed a cystic mass in the left nasal cavity, a proliferative lesion on the hard palate, and left eye proptosis with complete visual loss. Imaging demonstrated a mass arising from the left maxillary sinus with erosion of the orbital floor and optic nerve compression. Initial biopsy suggested a chondro-osseous respiratory epithelial hamartoma. Due to the lesion’s aggressive behavior and irreversible vision loss, the patient underwent left total maxillectomy with orbital exenteration and elective tracheostomy. Final histopathology confirmed conventional osteosarcoma, grade 3 (poorly differentiated/high-grade). Adjuvant chemotherapy with cisplatin and doxorubicin was administered. At the six-month follow-up, the patient remained disease-free, with no evidence of local recurrence or distant metastasis. Orbital extension of maxillary osteosarcoma is a rare and aggressive presentation. High clinical suspicion, comprehensive imaging, and histopathological confirmation are essential for diagnosis. Radical surgical resection, including orbital exenteration when indicated, combined with adjuvant chemotherapy, is critical for achieving oncologic clearance. Multidisciplinary management is key to improving outcomes in these complex cases.

## Introduction

Osteosarcoma, or osteogenic sarcoma, is a malignant bone tumor characterized by the production of osteoid or immature bone by neoplastic cells. Jaw osteosarcomas are rare, accounting for approximately 7% of all cases, and typically occur in adults between 30 and 50 years of age, with the mandible more frequently affected than the maxilla [1]. These tumors arise from mesenchymal tissue, with osteosarcoma and chondrosarcoma being the most common histological types, followed by Ewing’s sarcoma [2]. Clinically, patients often present with facial swelling, pain, and trismus [3].

Surgical resection with clear margins remains the cornerstone of treatment, often combined with adjuvant chemotherapy or radiotherapy. When tumors extend into the orbital contents, orbital exenteration (OE) may be required to achieve oncologically safe margins, despite significant functional and cosmetic consequences [4]. First described by George Bartisch in 1583, OE involves removal of the globe, extraocular muscles, periorbital fat, and frequently the eyelids, resulting in permanent disfigurement and potential psychological impact [5]. While OE is most commonly performed for eyelid and globe neoplasms [6], its use in maxillary osteosarcoma remains uncommon.

Given the rarity of orbital extension in maxillary osteosarcoma, the literature on management and outcomes is limited. This report describes a 41-year-old male with maxillary osteosarcoma involving the orbit, successfully treated with OE, highlighting clinical presentation, diagnostic evaluation, surgical strategy, postoperative outcomes, and considerations for multidisciplinary management in such rare cases.

## Case presentation

**Figure 1:**
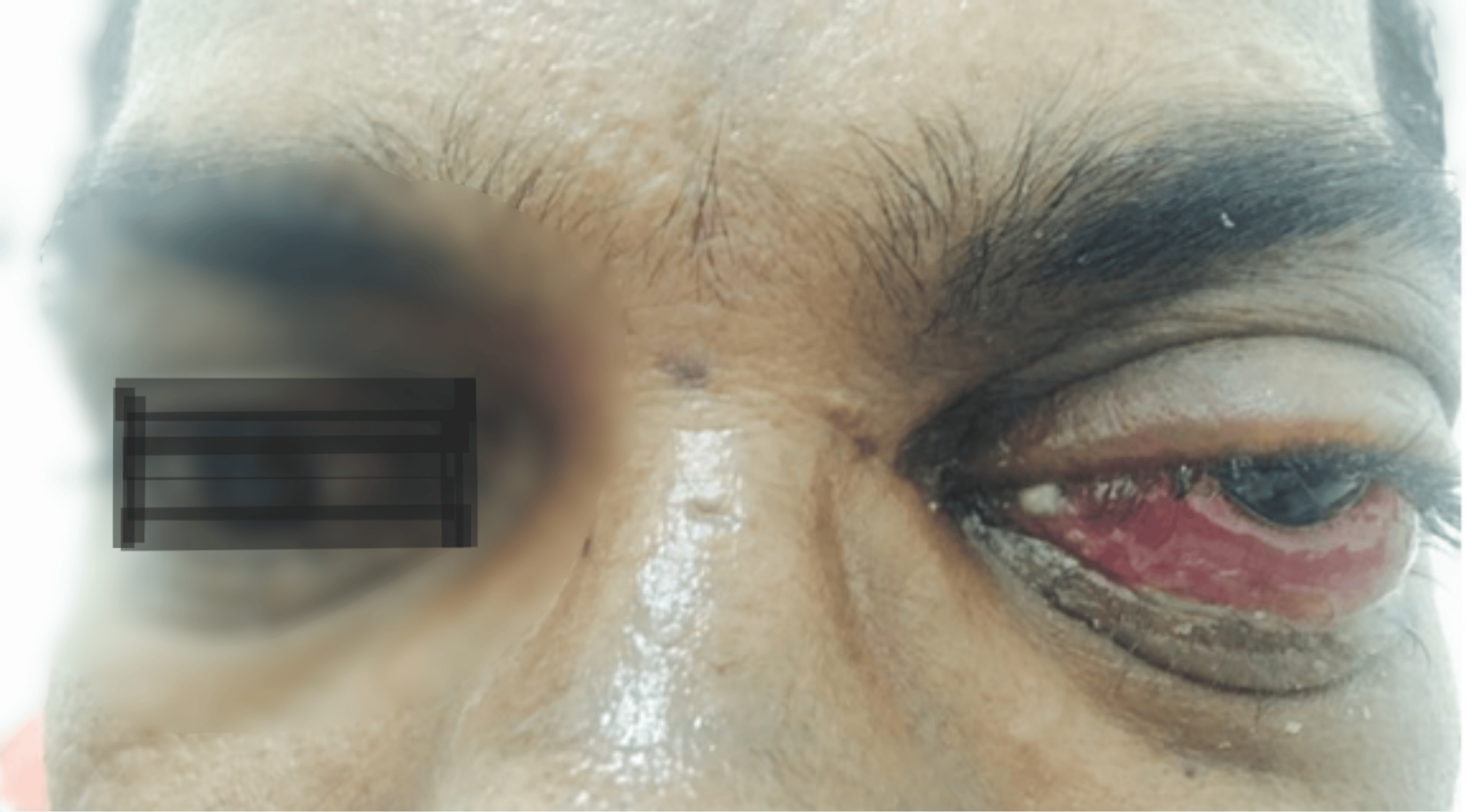
Preoperative clinical photograph demonstrating marked proptosis of the left eye, significant conjunctival congestion, chemosis, and swelling of the lower eyelid. The exposed conjunctiva appears inflamed and erythematous, indicating severe orbital involvement.

A 41-year-old male presented with a one-month history of progressive bilateral nasal obstruction and a 20-day history of vision loss in the left eye. He had been in good health until the onset of nasal obstruction, which was associated with occasional episodes of epistaxis and hyposmia. Over the subsequent weeks, he developed progressive proptosis of the left eye accompanied by swelling of the left hemiface (Figure 1). The patient had a recent diagnosis of type 2 diabetes mellitus and a 20-year history of smoking. There was no history of prior surgeries or other systemic illnesses.

**Figure 2:**
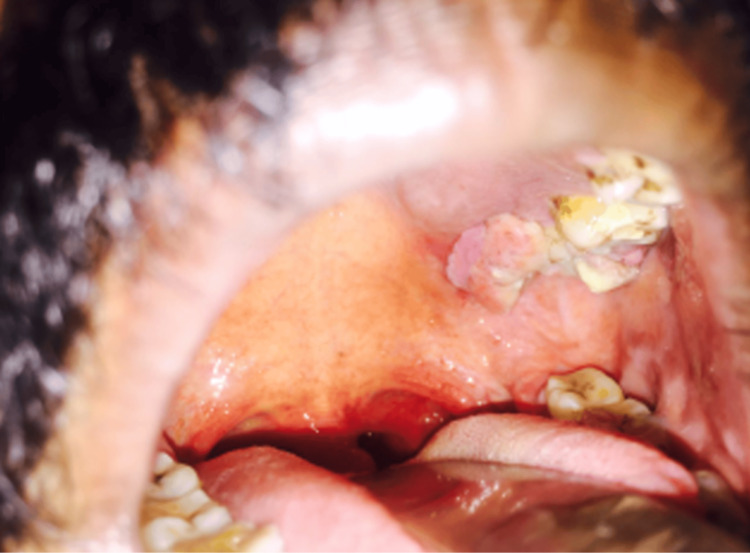
Intraoral examination revealing an ulceroproliferative mass involving the left maxillary alveolus and hard palate.

**Figure 3:**
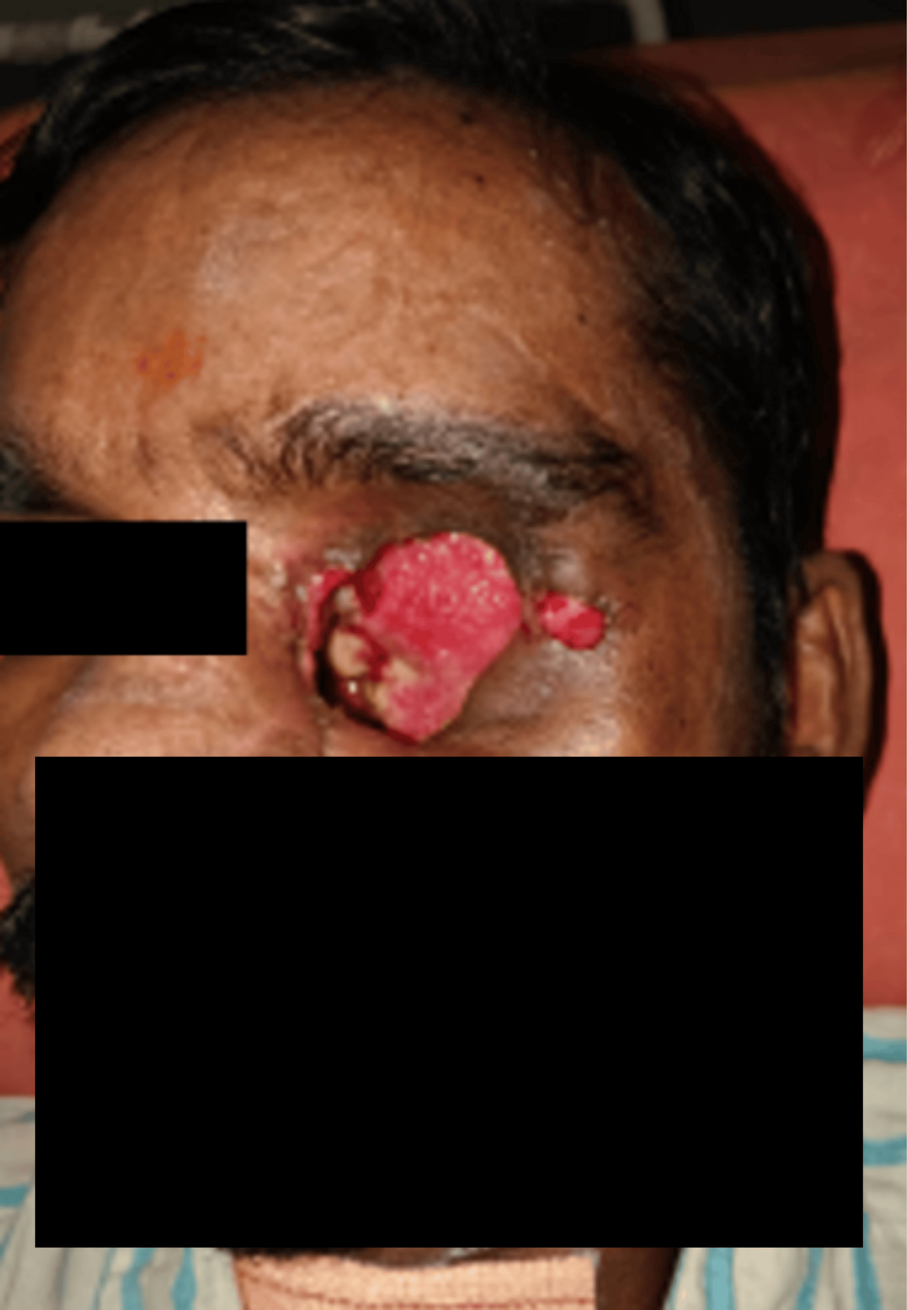
Advanced disease progression with extraorbital extension and fungating mass involving the left orbit prior to exenteration.

On clinical examination, a cystic, polypoidal mass occupied the left nasal cavity, displacing the septum toward the contralateral side. The mass bled on manipulation. Examination of the oral cavity revealed a proliferative lesion on the hard palate adjacent to the second and third molars (Figure 2). Ophthalmologic evaluation demonstrated left-sided proptosis with chemosis. Extraocular movements were absent, and the pupil was dilated with no pupillary reflexes or perception of light. Optical coherence tomography revealed macular detachment, and the optic disc appeared pale. Visual evoked potentials were absent, confirming optic nerve dysfunction (Figure 3).

Based on clinical and radiological findings, the differential diagnoses included rhino-orbital fungal infection, benign fibrous hyperplasia, and malignant sinonasal tumor.

**Figure 4:**
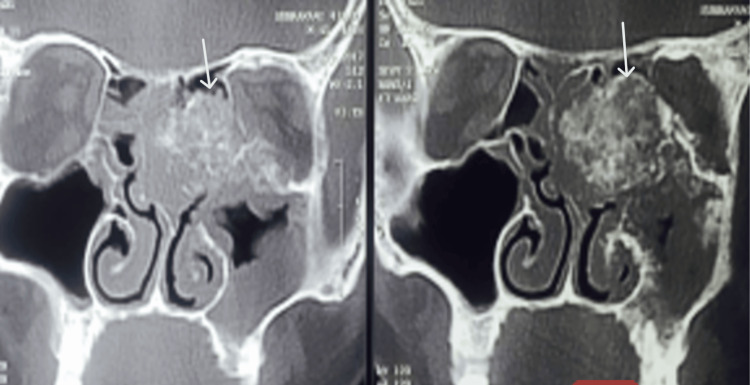
Contrast-enhanced computed tomography of the paranasal sinuses and orbit showing a heterogeneously enhancing mass arising from the left maxillary sinus with cortical destruction and superior extension into the orbital cavity. The tumor demonstrates bone erosion and soft tissue invasion involving the inferomedial orbital wall and adjacent maxillary structures.

Computed tomography of the paranasal sinuses and orbit revealed a mass arising from the left maxillary sinus, eroding the orbital floor and medial wall, and extending into the orbital soft tissues, resulting in proptosis and restricted ocular motility (Figure 4). Magnetic resonance imaging confirmed orbital invasion with optic nerve compression, correlating with the patient’s visual loss. The mass measured approximately 5.5 × 4.2 × 4.0 cm, with areas of osteoid matrix and coarse bony spicules. Adjacent structures, including the hard palate and nasal cavity, were partially involved, while the posterior maxillary wall remained intact.

An initial excision biopsy with endoscopic orbital decompression suggested chondro-osseous respiratory epithelial hamartoma. However, in view of malignant features on imaging and irreversible vision loss, the patient underwent left total maxillectomy with OE and elective tracheostomy. Final histopathological examination confirmed conventional osteosarcoma, grade 3 (poorly differentiated/high-grade), classified according to the WHO Classification of Tumours of Soft Tissue and Bone. [7] This discrepancy may be attributed to sampling error, intratumoral heterogeneity, or the presence of benign-appearing bone-forming features in small tissue samples. This highlights the importance of obtaining an adequate and representative biopsy combined with comprehensive imaging for accurate diagnosis of maxillary osteosarcoma. Microscopy demonstrated malignant spindle to polygonal cells producing osteoid, with marked pleomorphism and frequent mitotic figures. Surgical margins were tumor-free, and no perineural or lymphovascular invasion was identified.

Routine hematological and biochemical investigations were within normal limits, except for hyperglycemia secondary to diabetes mellitus. Chest radiograph and abdominal ultrasound showed no evidence of distant metastasis.

**Figure 5:**
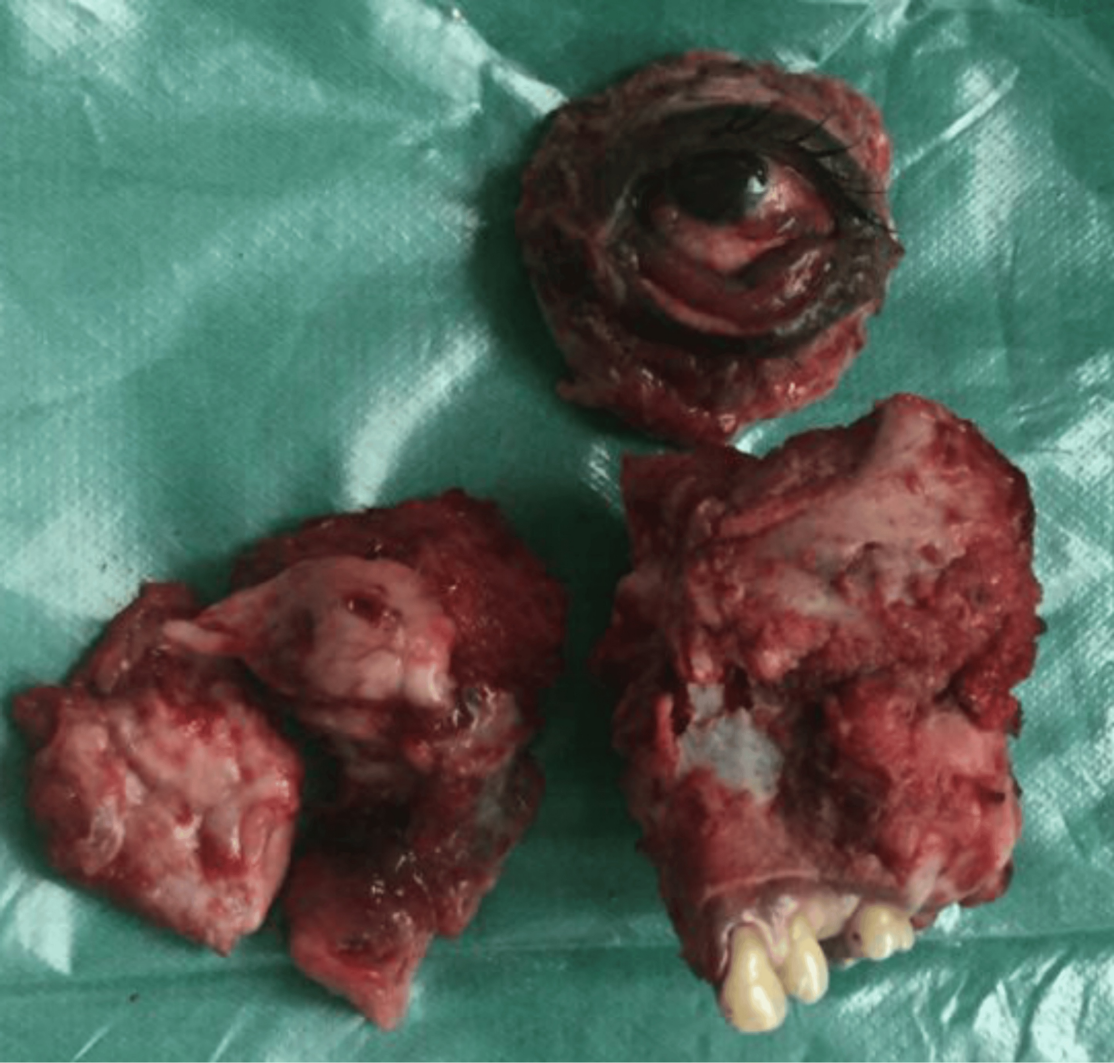
Gross specimen following left total maxillectomy with orbital exenteration showing the excised maxillary mass with attached teeth and the en bloc removal of the orbital contents. The specimen demonstrates tumor involvement of the maxillary sinus and orbital floor, consistent with extensive local invasion.

The patient’s symptoms progressed as follows: nasal obstruction began one month before presentation, and vision loss in the left eye developed twenty days prior to admission. Following the initial biopsy and decompression, definitive surgery was performed. Postoperative recovery was uneventful, and the surgical wound healed satisfactorily (Figure 5).

OE was indicated due to direct orbital invasion confirmed radiologically and intraoperatively, irreversible vision loss with optic nerve atrophy, and the need for complete oncologic clearance with negative surgical margins.

The patient was subsequently referred to the oncology unit and received adjuvant chemotherapy with a cisplatin- and doxorubicin-based regimen according to standard osteosarcoma protocols. Radiotherapy was not administered due to clear surgical margins. At the six-month follow-up, there was no evidence of local recurrence or distant metastasis, though long-term surveillance remains essential due to the aggressive nature of craniofacial osteosarcomas.

This case highlights the rare occurrence of orbital involvement in maxillary osteosarcoma and underscores the importance of early imaging, accurate histopathological diagnosis, and radical surgical intervention, including OE, for optimal oncologic control. A multidisciplinary approach involving otolaryngology, ophthalmology, pathology, and oncology teams was crucial in achieving a favorable clinical outcome.

## Discussion

Osteosarcoma of the maxilla is a rare malignancy, accounting for less than 10% of all head and neck osteosarcomas and under 1% of all osteosarcomas [8]. Its occurrence in the craniofacial skeleton poses unique diagnostic and therapeutic challenges due to the complex regional anatomy, proximity to vital neurovascular structures, and nonspecific early symptoms. Orbital involvement is exceptionally rare and typically reflects advanced disease with aggressive local invasion [4]. In this case, the tumor originated from the left maxillary sinus and extended into the orbit, resulting in proptosis and irreversible vision loss - a presentation reported in only a few cases [4,8].

Diagnosis of maxillary osteosarcoma is often delayed because the maxillary sinus allows significant tumor expansion before clinical detection. Early symptoms such as nasal obstruction, facial swelling, and epistaxis are frequently mistaken for benign sinonasal conditions [9]. In the present case, the initial biopsy suggested a chondro-osseous respiratory epithelial hamartoma, highlighting the diagnostic challenges and histopathological variability of these tumors. Therefore, obtaining an adequate, representative biopsy combined with comprehensive radiological evaluation is essential for accurate diagnosis and surgical planning.

Radiological imaging plays a pivotal role in assessing tumor extent, with features such as cortical destruction, mixed osteolytic-osteoblastic patterns, and soft tissue or orbital invasion suggesting malignancy. Histopathological confirmation remains the gold standard, and tumor grade strongly correlates with prognosis [10]. The present patient had a high-grade (grade 3) conventional osteosarcoma, warranting aggressive surgical management.

OE is indicated when direct orbital invasion occurs or vision is irreversibly lost, to achieve complete oncologic clearance and minimize the risk of local recurrence [11]. Cumming et al. [12] reported outcomes of 39 patients undergoing OE for peri-orbital or orbital malignancies, emphasizing that timely radical surgery provides the best chance for disease control. Pain and swelling are the most common presenting features, as observed in our patient [13].

Complete surgical excision with tumor-free margins remains the cornerstone of curative treatment in craniofacial osteosarcoma. Adjuvant chemotherapy with cisplatin and doxorubicin, as administered in this case, has been shown to improve disease-free survival when combined with complete resection.

Given the rarity and complexity of maxillary osteosarcoma with orbital extension, multidisciplinary management involving otolaryngology, ophthalmology, maxillofacial surgery, pathology, and oncology is imperative. Such collaboration ensures accurate diagnosis, comprehensive treatment, and optimal functional and aesthetic outcomes. At the six-month follow-up, the patient demonstrated no local recurrence or distant metastasis, highlighting the importance of early radical intervention and vigilant postoperative surveillance. Long-term follow-up remains essential, as local recurrence continues to be the primary determinant of prognosis in craniofacial osteosarcomas.

## Conclusions

Orbital involvement in maxillary osteosarcoma is a rare and aggressive entity that poses significant diagnostic and therapeutic challenges. This case highlights the importance of early recognition of progressive nasal obstruction, facial swelling, and ocular symptoms, supported by thorough radiological and histopathological evaluation for accurate diagnosis and staging. Radical surgical intervention, including OE, may be warranted in cases with direct orbital invasion and irreversible vision loss to achieve complete oncologic clearance. Multidisciplinary management is essential to optimize outcomes, minimize complications, and improve overall survival. Careful individualized treatment planning and vigilant long-term follow-up are crucial in managing such rare cases.
